# Seasonal variation of Cesium-137 concentration in Asian black bear (*Ursus thibetanus*) and wild boar (*Sus scrofa*) in Fukushima Prefecture, Japan

**DOI:** 10.1371/journal.pone.0200797

**Published:** 2018-07-18

**Authors:** Yui Nemoto, Rie Saito, Hitoshi Oomachi

**Affiliations:** Research Department, Fukushima Prefectural Centre for Environmental Creation, Miharu Town, Fukushima, Japan; University of South Carolina, UNITED STATES

## Abstract

To elucidate and reduce the risk of radionuclide contamination in wildlife caused by the Tokyo Electric Power Company Fukushima Dai-ichi Nuclear Power Station accident, it is important to understand radionuclide variations in the wild animal population. Here, we used environmental monitoring data and muscle samples collected from Asian black bear (*Ursus thibetanus*) and wild boar (*Sus scrofa*) from May 2011 to March 2016 to examine seasonal variation in radiocesium (^137^Cs) concentrations in muscle tissues (hereafter, muscle ^137^Cs) of these important game species in Fukushima Prefecture. We measured muscle ^137^Cs of bears and wild boars killed by hunters or in animal control culls. First, using a linear mixed model (LMM), we tested for a positive relationship between muscle ^137^Cs and ^137^Cs in the soil at the site of capture (hereafter, soil ^137^Cs) estimated from a soil ^137^Cs deposition map produced by the Japan Atomic Energy Agency. In the LMM, muscle ^137^Cs was positively related to estimated soil ^137^Cs, which corroborates the results of previous studies. The LMM regression coefficients differed between the two species, with wild boar muscle ^137^Cs being higher than that of bears sampled at the same locations. We then employed a generalized additive mixed model (GAMM) to estimate seasonal variation in the muscle ^137^Cs of the target species. GAMM showed that muscle ^137^Cs varied seasonally and that this seasonal variation also differed between the two species. In bears, muscle ^137^Cs decreased from spring to early autumn, before increasing in winter. Wild boar muscle ^137^Cs remained low during spring and summer and was high during autumn and early spring. These patterns are likely influenced by differences in diet, habitat use, and physiology between these two species.

## Introduction

The Great East Japan Earthquake on March 11, 2011 caused severe damage to the Tokyo Electric Power Company Fukushima Dai-ichi Nuclear Power Station (FDNPS) and large amounts of radionuclides (e.g. 90-700PBq of ^131^I and 7-50PBq of ^137^Cs) were emitted into the environment [1]. The radionuclides, especially ^134^Cs and ^137^Cs, were transported to various ecosystems and were detected in wildlife living near the FDNPS [2,3]. Radionuclides in wildlife have harmful effects at the individual and population levels through internal radiation exposure [4,5], and there is a further risk of radionuclides in game animals being transferred to humans through consumption of game species [3,6,7]. Thus, to elucidate and prevent the risk of radionuclide contamination in wildlife and transfer to humans, it is important to understand the distribution and variation of radionuclides in wildlife species.

In Europe after the Chernobyl Nuclear Power Station accident (April 26 1986), the ^137^Cs activity concentration in wild boar was reported to be higher than in other animals, and the elevated levels were suggested to be due to the consumption of fungus [8]. High concentrations of radiocesium were also recorded in wild boar (*Sus scrofa*) in Japan after the FDNPS accident [3]. However, there is no evidence that wild boar forage on fungus [9–11], and the reason for the high radionuclide concentration in wild boar in Japan is unknown.

Because the Asian black bear (*Ursus thibetanus*) is an equally important game species as wild boar in eastern Japan where the impacts of the FDNPS accident were greatest, elucidation of the radionuclide transfer mechanisms and prediction of radionuclide concentration patterns in bears is needed. Radiocesium concentrations in bears were lower than in wild boar after the FDNPS [3], even though both bears and wild boar are omnivores [12]. It is important to demonstrate why radiocesium concentration differs between bears and wild boar. However, few studies on radionuclide transfer involving bear species have been conducted [13,14].

In a previous study examining the effects of the Chernobyl Nuclear Power Station accident on wild animals, variations in ^137^Cs activity concentrations between individuals and between years in the same wild animal species were shown to be strongly affected by biological factors such as changes in food habits and movement patterns extending until about 30 years after the accident [15]. In the wild animals in Europe, ^137^Cs activity concentrations were shown to be elevated in seasons when the animals ate highly contaminated food items [8,16,17]. Thus, understanding seasonal variation in radionuclide concentrations in wild animals is important to determine the relationship between wild animal ecological traits and radionuclide concentrations. Nevertheless, no studies on the seasonal variation in radionuclide concentrations in Japanese wild animals in the region impacted by the FDNPS accident have been conducted.

In this study, we focused on seasonal variation in ^137^Cs concentration in muscle, which is strongly related to biological factors. We investigated the seasonal variation in radionuclide concentrations in the muscle of Asian black bear and wild boar and compared the variation between the two target species. We selected ^137^Cs in the muscle of wild animals as the radionuclide of interest because it has a longer half-life (30.1 years) and a greater tendency to influence the organism through internal accumulation than the other radionuclides (^131^I and ^134^Cs) that were emitted in large amounts during the FDNPS accident [18,19]. In addition, because ^137^Cs activity in wild animals is positively affected by the ^137^Cs ground deposition density in the soil at the site of capture [6,20–22], we investigated the relationship between ^137^Cs activity concentration in the muscle and the soil at the site of capture before analyzing seasonal variation. Then, we analyzed the seasonal variation in ^137^Cs activity concentration in the muscle tissue of bear and wild boar while taking ^137^Cs ground deposition density in the soil at the site of capture into consideration. In Europe, ^137^Cs concentration in the muscle of wild animals was high in seasons when fungi and underground food items accounted for a greater proportion of the diet [8,16,17], and it was lower in seasons with a higher availability of hard masts for food items [23–25]. Thus, we set the following hypotheses regarding seasonal variation in ^137^Cs activity concentration in the muscle: 1) ^137^Cs concentration in the muscle of wild boar would be high in winter and relatively low in spring to autumn because they forage underground food such as roots in winter [9–11], and 2) ^137^Cs concentration in the muscle of Asian black bear would be low in autumn to winter and relatively high in summer because they forage hard masts mainly in autumn to winter [12,26,27]. In previous study, ^137^Cs concentration in the muscle of wild animals was high when the amount of food intake was high [17]. Asian black bear consumes large amounts of hard masts in autumn to prepare for winter denning. In addition, bear species do not excrete during winter denning [28] and the suppression of excretion might be accelerate the accumulation of ^137^Cs in their bodies. Therefore, we added the following hypothesis: 3) ^137^Cs concentration in the muscle of Asian black bear would be higher in autumn to winter than in summer.

## Materials and methods

### ^137^Cs activity concentration in wild animals

In this study, we obtained 279 muscle samples from carcasses of Asian black bear and 1,033 muscle samples from carcasses of wild boar that were killed by hunters or in animal control culls kills in Fukushima Prefecture from May 2011 to March 2016. Each sample was at least 500 g and taken from the thigh of the killed animal. We recorded the capture date and site (latitude, longitude) for each animal. After mincing the sample and extracting the fat and connective tissue, the samples were transferred to U-8 vessels (100 ml). We followed all guidelines for the ethical use of animals in research by The Mammal Society of Japan [29].

**Fig 1:**
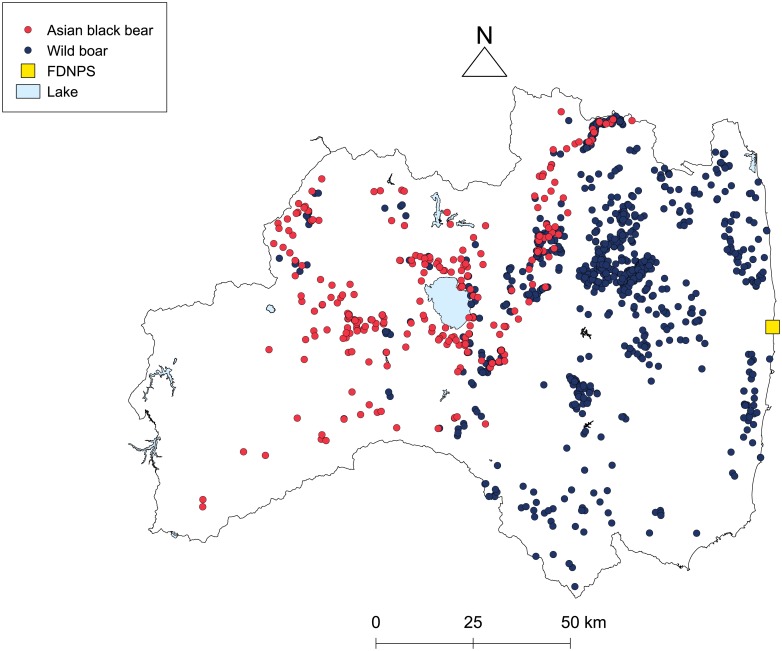
Capture sites of Asian black bear and wild boar in Fukushima Prefecture that were included in the analysis. The red and blue circles represent the capture sites of Asian black bear and wild boar, respectively. The yellow square indicates the FDNPS. The border of Fukushima Prefecture and lakes were drawn based on data from the Ministry of Land, Infrastructure, Transport and Tourism [30].

The ^137^Cs activity concentration (Bq/kg fresh matter [FM]) in each muscle tissue sample (hereafter, muscle ^137^Cs) was measured at the Radiation Survey Division of Fukushima Prefectural Government using a high-purity germanium detector (GC3018, GC4020, and GR4521 Canberra Japan, Tokyo, Japan) with the count time set to 3,600 s and the detection limit set to 4–9 Bq/kg. We calculated the muscle ^137^Cs value on the animal capture date for each sample using the physical half-life of ^137^Cs (30.1 years). For statistical analysis, we used muscle ^137^Cs value samples for each species for which ^137^Cs concentration was above the detection limit (271 Asian black bear samples and 1,031 wild boar samples) and the geographic location of each capture site (Fig 1).

### ^137^Cs ground deposition data at site of capture

To consider the ^137^Cs ground deposition in the soil at the site of capture (hereafter, soil ^137^Cs) for analysis of seasonal variation in the muscle ^137^Cs for each species, we estimated soil ^137^Cs (Bq/m^2^) based on the ^137^Cs ground deposition map of the Japan Atomic Energy Agency’s (JAEA) 5th Airborne Monitoring Survey, which covered all of Fukushima Prefecture [31]. The muscle ^137^Cs of animals is influenced more by factors of the area surrounding the capture site than by those of the home range area because of their large scale movement [20]; therefore, we used the soil ^137^Cs value at the capture site in the statistical analysis. For statistical analysis, we also calculated the soil ^137^Cs value on the capture date for each sample using the physical half-life of ^137^Cs (30.1 years) and the time difference (days) between the capture date and the survey date of the ^137^Cs ground deposition map (June 28, 2012). We used QGIS 2.16.1 [32] to extrapolate the soil ^137^Cs value at the capture site.

We calculated the total dose rate for Asian black bear and wild boar using our data of muscle ^137^Cs and soil ^137^Cs using the ERICA tool [33–35]. We transformed soil ^137^Cs (Bq/m^2^) into ^137^Cs activity concentration in soil (Bq/kg) using the following formula: Bq/kg = Bq/m^2^/([area: 100 × 100 cm × depth: 5 cm × bulk density: 0.9 g/ cm^3^]/1,000 g/kg). For the transformation formula, we used the depth of soil at which most ^137^Cs is distributed (5 cm) and the bulk density of soil (0.9 g/m^3^) from a previous report [36]. In the calculation, we used the following body measurements of Asian black bear and wild boar from previous reports in the Kanto region of Japan [37,38]: mass, 52.75 kg; body height, 0.68 m; body width, 0.68 m; and body length, 1.25 m for Asian black bear; mass, 75 kg; body height, 0.68 m; body width, 0.68 m; and body length, 1.10 m for wild boar. Since the body height and width of Asian black bear have not been previously reported, we used the values from a previous report on wild boar [37]. Default values of Tier 2 in the ERICA tool were used for other factors of the calculation.

### Statistical analysis

Before analyzing the seasonal variation, we analyzed the relationship between muscle ^137^Cs and soil ^137^Cs at the capture site using the Linear Mixed Model (LMM). In the LMM, we used muscle ^137^Cs as the response variable and soil ^137^Cs as the explanatory variable to estimate the relationship between muscle ^137^Cs and soil ^137^Cs. To show the difference in the relationship by species, we used animal species (Asian black bear or wild boar) as an interaction term of the explanatory variable. Sampling year was used as a random factor to estimate pure relationships between muscle ^137^Cs and soil ^137^Cs without environmental fluctuation among years (e.g. the annual fluctuation of hard mast production, which affect the behavior of the target species) [10,39–44]. The model selection of LMM was operated by Akaike information criterion (AIC), and informative models were defined as models with delta AIC < 2.0.

In the next stage, we analyzed seasonal variation in muscle ^137^Cs using the Generalized Additive Mixed Model (GAMM). In the GAMM, we used muscle ^137^Cs as the response variable and capture month (integer data starting in April [month 1] and ending in March [month 12] because the sampling started in April each year) as the explanatory variable to estimate seasonal variation in muscle ^137^Cs. Animal species (Asian black bear or wild boar) was used as an interaction term of the explanatory variable to show statistical differences in the seasonal variation between the two species. We used sampling year as a random factor, for the same reason given for the LMM above. We removed the effect of positive relationships between muscle ^137^Cs and soil ^137^Cs on seasonal variation in muscle ^137^Cs by using soil ^137^Cs as the offset term to estimate pure seasonal variation in muscle ^137^Cs. Because the seasonal variation showed a cyclic pattern, we used the cyclic cubic spline smooth as the smoothing term, and estimated the degree of smoothing using the restricted maximum likelihood method [45]. Then, we tested the GAMM selection by AIC, and defined models with delta AIC < 2.0 as informative models.

In the statistical analyses, we used muscle ^137^Cs and soil ^137^Cs with log_10_ transformation. We used R3.3.1 [46], mgcv package [45], lme4 package [47], and MuMIn package to estimate LMM and GAMM.

## Results

### Relationship between soil and muscle ^137^Cs

In Asian black bear, the range of muscle ^137^Cs and soil ^137^Cs was 4–1,080 Bq/kg FM and 1,563–227,118Bq/m^2^, respectively (Table 1). The range of the aggregated transfer factor, *T*_*ag*_ (m^2^kg^-1^FM: muscle ^137^Cs / soil ^137^Cs) for Asian black bear was 2.2×10^−4^–5.3×10^−2^ m^2^kg^-1^FM. In wild boar, the range of muscle ^137^Cs and soil ^137^Cs was 7–40,200 Bq/kg FM and 4,691–1,135,776 Bq/m^2^, respectively. The range of *T*_*ag*_ for wild boar was 9.2×10^−5^–9.1×10^−1^ m^2^ kg^-1^FM.

**Table 1:** ^137^Cs concentration in muscle and soil at capture site and *T*_ag_ for Asian black bear and wild boar in Fukushima Prefecture.

Species	Muscle ^137^Cs (Bq/kg FM)				n	Soil ^137^Cs (Bq/m^2^)				*Tag* (m^2^kg^-1^FM)			
	Mean	SD	Min	Max		Mean	SD	Min	Max	GM	GSD	Min	Max
Asian black bear	100	123	4	1080	271	45,853	48,728	1,563	227,118	2.2×10^−3^	2.3	2.2×10^−4^	5.3×10^−2^
Wild boar	900	2,743	7	40,200	1,031	129,919	131,286	4,691	1,135,776	3.2×10^−3^	3.4	9.2×10^−5^	9.1×10^−1^

FM, fresh matter; *T*_ag_, aggregated transfer factor; SD, standard deviation, GM, geographic mean, GSD, geographic standard deviation

Using the ERICA tool, the mean and the range of total dose rate of Asian black bear was 0.103 μGy/h and 0.007–0.477 μGy/h, respectively. In wild boar, the mean and the range of total dose rate was 0.479 μGy/h and 0.010–12.700 μGy/h, respectively.

**Fig 2:**
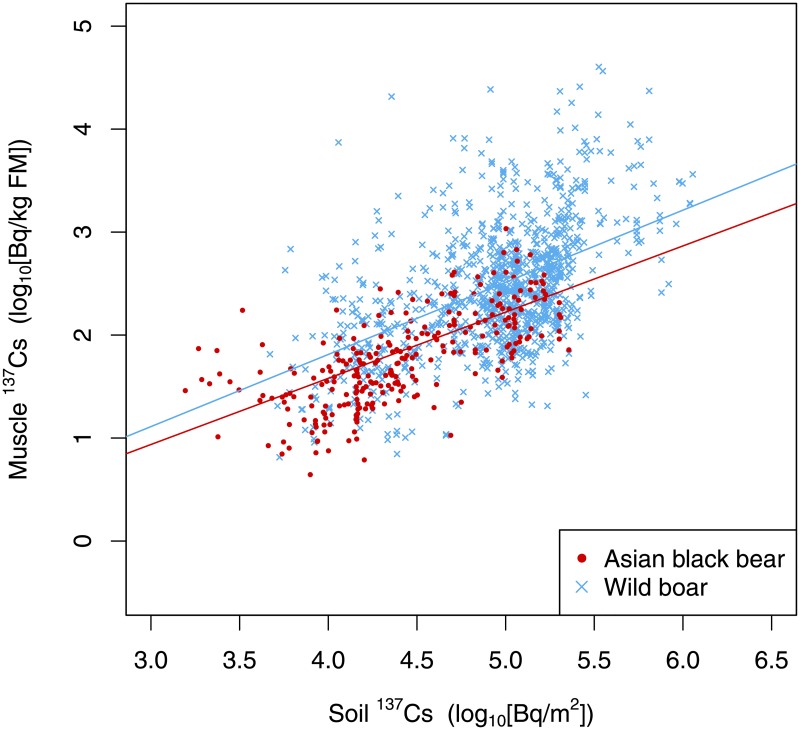
Relationship between muscle ^137^Cs and soil ^137^Cs at the capture site in Asian black bear and wild boar. Red and blue lines represent the LMM regression lines for Asian black bear (y = 0.643 × x − 0.991) and wild boar (y = 0.701 × x − 0.991), respectively.

Application of the LMM for both target species demonstrated a positive relationship between muscle ^137^Cs and soil ^137^Cs (Table 2, Fig 2). Further, the LMM selected based on delta AIC < 2.0 included animal species as the interaction term of the explanatory variable (Table 2). This result shows that the relationship between muscle ^137^Cs and soil ^137^Cs differed for Asian black bear and wild boar. For the same soil ^137^Cs level, muscle ^137^Cs was higher for wild boar than for Asian black bear (Fig 2).

**Table 2:** LMM selected by AIC.

Model No.	Formula	AIC	Delta AIC
1	Muscle ^137^Cs (log_10_[Bq/kg FM]) = Soil ^137^Cs (log_10_[Bq/m^2^]): Species^a^ + Intercept	1730.20	0.00*
2	Muscle ^137^Cs (log_10_[Bq/kg FM]) = Soil ^137^Cs (log_10_[Bq/m^2^]) + Intercept	1773.62	43.42
3	Muscle ^137^Cs (log_10_[Bq/kg FM]) = Species^a^ + Intercept	2165.79	435.59
4	Muscle ^137^Cs (log_10_[Bq/kg FM]) = Intercept	2385.00	654.80

^a^Species had two categorical values (Asian black bear and wild boar).

*: The informative model which was defined as models with delta AIC < 2.0.

### Seasonal variation in muscle ^137^Cs

**Fig 3:**
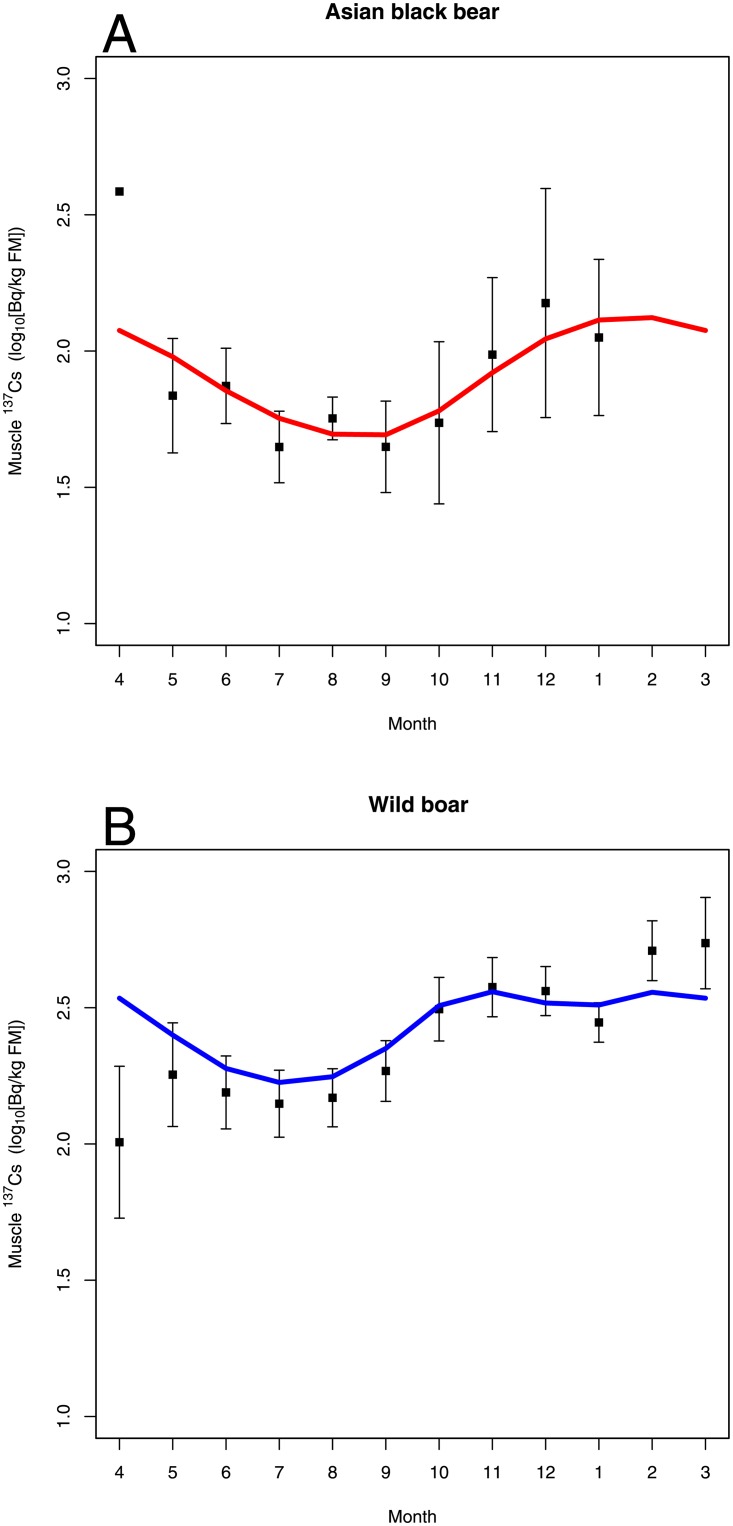
Seasonal variation in muscle ^137^Cs for (A) Asian black bear and (B) wild boar. The boxes and bars indicate the mean and 95% confidence intervals, respectively. The curves are regression curves obtained from GAMM analysis, which used the mean soil ^137^Cs value of each species as the offset.

Application of GAMM to analyze seasonal variation in muscle ^137^Cs in Asian black bear and wild boar identified two models based on AIC that both included capture month as an explanatory variable (Table 3). Thus, muscle ^137^Cs was shown to vary by season. A GAMM selected based on AIC included animal species as an intercept term of the explanatory variable (Table 3). Thus, the seasonal variation pattern differed between Asian black bear and wild boar. The regression curves in Fig 3 for muscle ^137^Cs obtained from the GAMM including capture month and animal species showed that; muscle ^137^Cs of Asian black bear showed a decrease from May to September, followed by an increase from October to January. In wild boar, muscle ^137^Cs was low from April to August, increased from September to November, and was high from December to March.

**Table 3:** GAMM selected by AIC.

Model No.	Formula	AIC	Delta AIC
1	Muscle ^137^Cs (log_10_[Bq/kg FM]) = s(Month^a^): Species^b^ + offset (Soil ^137^Cs (log_10_[Bq/m^2^])) + Intercept	1759.80	0.00*
2	Muscle ^137^Cs (log_10_[Bq/kg FM]) = s(Month^a^) + offset (Soil ^137^Cs (log_10_[Bq/m^2^])) + Intercept	1760.48	0.68*
3	Muscle ^137^Cs (log_10_[Bq/kg FM]) = Species^b^ + offset (Soil ^137^Cs (log_10_[Bq/m^2^])) + Intercept	1821.48	61.68
4	Muscle ^137^Cs (log_10_[Bq/kg FM]) = offset (Soil ^137^Cs (log_10_[Bq/m^2^])) + Intercept	1838.90	79.10

^a^Month is capture month of the individual animal.

^b^Species had two categorical values (Asian black bear and wild boar).

*: The informative model which was defined as models with delta AIC < 2.0.

## Discussion

### Relationship between soil ^137^Cs and muscle ^137^Cs

Our study demonstrated a positive relationship between muscle ^137^Cs and soil ^137^Cs in Asian black bear and wild boar at each capture site. This result supports the findings of previous studies showing that radionuclide concentration in muscle was high in animals sampled from areas with high radionuclide ground deposition or high radioactive contamination level [6,20–22]. Thus, determination of radioactive contamination levels or radionuclide ground deposition at sampling sites seems to be important for analyzing radionuclide concentration in wildlife sampled from a large study area. However, muscle ^137^Cs was highly varied among animals captured in areas with similar soil ^137^Cs levels. As shown in Fig 2, muscle ^137^Cs varied by one order of magnitude among Asian black bears that were captured in areas with similar soil ^137^Cs. In wild boar, muscle ^137^Cs varied by three orders of magnitude in areas with similar soil ^137^Cs. These variations suggest the importance of accounting for other factors such as season in order to understand the mechanism of radionuclide concentration in wild animals.

In our study, the muscle ^137^Cs of wild boar was higher than that of bears sampled at the same location. In Europe, the muscle ^137^Cs of wild boar was higher than that of ungulate species because wild boar in Europe foraged food with high ^137^Cs such as fungus and food items found underground [7,8]. In Japan, wild boar are not known to forage fungus but they do frequently forage for food items found underground such as roots and tubers [9–11]. Asian black bear in Japan have been found to forage ant species found underground [12,26,27] and rarely feed on fungi and tubers [12]. Therefore, we suppose that the higher muscle ^137^Cs in wild boar is a consequence of frequent foraging on roots and tubers compared to a much lower inclusion of these food items in the diet of the bear. To further identify the reasons for higher muscle ^137^Cs in wild boar, examination of ^137^Cs concentration in individual food items in the diet of each animal is needed.

When we compared the *T*_*ag*_ of Asian black bear in Fukushima Prefecture after the FDNPS accident with that of brown bear in Europe after the Chernobyl Nuclear Power Station accident, the geographic mean of Asian black bear (2.2×10^−3^ m^2^kg^-1^FM) in Fukushima Prefecture was lower than that of brown bear (7.0 x 10^−2^ m^2^kg^-1^FM) in Finland [13]. The same trend was found in wild boar; the geographic mean of *T*_*ag*_ from wild boar in Fukushima Prefecture after the FDNPS accident (3.2×10^−3^ m^2^kg^-1^FM) was lower than that in Europe after the Chernobyl Nuclear Power Station accident (8.0×10^−3^ m^2^kg^-1^FM in 2003 and 6.2×10^−2^ m^2^kg^-1^FM in 2004) [25]. However, the range of *T*_*ag*_ from wild boar in Fukushima Prefecture (9.2×10^−5^ to 9.1×10^−1^ m^2^kg^-1^FM) was larger than that in Europe (4.0×10^−3^ to 1.5×10^−1^ m^2^kg^-1^FM) [8]. These differences also might be influenced by food habits differences between Japan and Europe. For example wild boar in Europe forage fungus whereas wild boar in Japan do not. Because these studies had different protocols for sampling animal meat and soil at capture site, we need to compare radiocesium contamination of wild animals in Fukushima Prefecture and Europe using a unified method to elucidate the factor effect on the radiocesium transfer from environment to wild animal.

When we calculated the total dose rate from our data using the ERICA tool [33–35], total dose rate ranges of Asian black bear and wild boar in Fukushima Prefecture between 2011 and 2016 were 0.007–0.477 μGy/h and 0.010–12.700 μGy/h, respectively. From the ERICA tool database, minor decreases in body weight and moderate decreases in population density were mentioned for otter species in dose rate, similar to the maximum value of wild boar, however, radiological risk associated with the dose rate range of Asian black bear was not mentioned [33–35]. However, caution should be exercised when using these values because we used body geometries of both animal species from other regions [37,38] for the total dose rate calculation in the ERICA tool. Specially, we used the body height and body width of wild boar as a substitute for body measurements of Asian black bear because these values have not been reported for Asian black bear. In addition, this evaluation did not consider temporal and spatial differences in habitat use by animals that might have been affected on dose rate [48]. To accurately evaluate the total dose rate of these species, body measurement, particularly mass, body length, body height, and body width of the target species in the study region and consideration of habitat use by the target species is needed.

### Seasonal variation in muscle ^137^Cs

Our study revealed that muscle ^137^Cs of wild boar and Asian black bear varied by season, and the pattern of seasonal variation differed by species. In Europe, seasonal variation in muscle ^137^Cs was observed in roe deer (*Capreolus capreolus*) and wild boar, and muscle ^137^Cs of those species was high from summer to autumn when fungi and underground food items accounted for a greater proportion of the diet [8,16,17], and it was lower in seasons and in regions with a higher availability of hard masts for food items [23–25]. On the other hand, seasonal variation in muscle ^137^Cs was not observed in red deer (*Cervus elaphus*), which did not forage fungi [8]. Therefore, it is possible that seasonal variation in muscle ^137^Cs in wild animals is affected by the food habits of the animals. In wild boar in Fukushima, muscle ^137^Cs was low from spring to summer and high from autumn to winter. This seasonal variation pattern supported our hypothesis 1, but it differed from seasonal patterns observed in Europe. Wild boar in Japan mainly forage the leaves of grasses and woody species in spring and summer, and mainly forage roots, tubers and hard masts in autumn and winter [9–11]. Thus, we expected that the consumption of roots and tubers would result in high muscle ^137^Cs in wild boar in Fukushima. However, because wild boar are omnivores, their food habits change with changing habitat conditions, resulting in seasonal variation patterns in muscle ^137^Cs. For example, when hard masts are abundant, muscle ^137^Cs decreases during part of the peak season of muscle ^137^Cs because wild boar forage large amount of hard masts in autumn [23,25]. Further, studies comparing ^137^Cs concentration in food items in the diets of wild animals in Japan are lacking. Further study of the seasonal food habits of wild boar in Fukushima and ^137^Cs concentrations in food items are needed to further elucidate the reasons for seasonal variations in muscle ^137^Cs.

In Asian black bear, muscle ^137^Cs increased from autumn to winter. This result did not support our hypothesis 2, but our hypothesis 3 was supported. The autumn and winter seasons coincide with large intakes of hard masts and denning [12,26,27]. Because previous studies of wild boars reported that muscle ^137^Cs was low when foraging on hard masts was higher because hard masts contained low ^137^Cs [23–25], factors other than ^137^Cs concentration in food items are likely to be responsible for the elevated muscle ^137^Cs of Asian black bear. The muscle ^137^Cs of roe deer increased during the season in which food intake was higher [17]. Therefore, we should not only study food habit and ^137^Cs concentration in food items, but also the effects of intake rate on seasonal variation in muscle ^137^Cs of the Asian black bear. However, the food habits of the Asian black bear in Fukushima and ^137^Cs concentration in food items has not been reported, and these remain important issue for understanding the seasonal variation in muscle ^137^Cs.

Our hypothesis 3 was supported by the study results, however, understanding the physiological characteristics of the target species is also important to revealing the mechanism of seasonal variation in radionuclide concentrations. Particularly for Asian black bear, muscle ^137^Cs increased in autumn when bears prepared for denning and peaked in the winter denning season. Other bear species such as the American black bear (*U*. *americanus)* and brown bear (*U*. *arctos*) also have a denning season in winter [49], and the American black bear does not urinate or void during this period [28]. Suppression of evacuation in the denning season probably accelerates the accumulation of ^137^Cs, and physiological changes in bear species in preparation for denning begin to take place in autumn [50,51]. Thus, if the physiological changes in the denning season of Asian black bear are the same as those or other bear species, these changes may result in an increase in muscle ^137^Cs from autumn to winter. To understand the mechanisms of seasonal change in muscle ^137^Cs in bear species, understanding physiological changes during the denning season is also important.

The muscle ^137^Cs of Asian black bear changed most drastically during autumn when they mainly foraged on hard masts and had the lowest muscle ^137^Cs in September followed by the highest muscle ^137^Cs of the year in November and December. It is possible that changes in the location of foraging as hard masts drop from treetops to the ground during late autumn may play a role in the increase in muscle ^137^Cs of Asian black bear. In the forest environment, radionuclides are more heavily concentrated at the forest floor, which is the habitat of the Asian black bear and wild boar [52,53]. Thus, it is possible that intake of radionuclides increased when the bears foraged hard masts on the ground through foraging soil and litter along with the hard masts. To reveal the mechanism of the changes in muscle ^137^Cs in Asian black bear in autumn, it is necessary to show the relationships between intake of radionuclides and changes in foraging location. Recently, bio-logging technology (e.g. Global Position System collar and accelerometer) to monitor animal behavior has advanced and the application of such technology to study the relationships between radionuclide intake and animal behavior, especially changes in foraging location, is promising.

In this study, no data were obtained for muscle ^137^Cs of Asian black bear during February to March, which is after the end of the hunting season on February 15 and when bears enter denning. Obtaining muscle samples during this period will be difficult but important to elucidating the relationships between radionuclide concentrations in the muscle of the Asian black bear and physiological changes due to denning.

## Conclusions

This is one of the few studies to report seasonal variation in radionuclide concentration in wild animals after the FDNPS accident. Notably, to the best of our knowledge, this is the first report on seasonal variation in radionuclide concentration in a bear species. The findings of our study provide useful information not only about the radionuclide dynamics of wild animals but also monitoring radionuclide concentration in wild animals in Japan. Such information may be helpful in developing hunting management strategies based on monitoring. In Fukushima Prefecture, transport of Asian black bear and wild boar was restricted following the report of muscle ^137^Cs exceeding the standard limits for general foodstuffs (100 Bq/kg). From our findings, sampling for radiocesium monitoring during winter when was the muscle ^137^Cs is likely to be at its maximum will be necessary to lift the transportation restriction. Radiocesium monitoring in Asian black bear and wild boar that excludes winter sampling would result in underestimation, and we therefore need to develop methods for radionuclide concentration monitoring that take into consideration the seasonal variation in radionuclide concentrations in wild animals.

Our study showed that radionuclide concentrations and seasonal variation differed between two large omnivorous mammal species in a region impacted by radionuclide contamination. To elucidate the mechanism of radionuclide transfer from the environment to wild animals, it is necessary to study factors that affect the specific differences that were revealed in our study. Taking concepts from previous studies and the ecological traits of both species into consideration, we suggest that the following factors are the most important when evaluating a target species: 1) food habits, 2) habitat use, and 3) seasonal changes in physiology.
